# Visceral Obesity and Metabolic Syndrome Are Associated with Well-Differentiated Gastroenteropancreatic Neuroendocrine Tumors

**DOI:** 10.3390/cancers10090293

**Published:** 2018-08-27

**Authors:** Ana P. Santos, Ana C. Santos, Clara Castro, Luís Raposo, Sofia S. Pereira, Isabel Torres, Rui Henrique, Helena Cardoso, Mariana P. Monteiro

**Affiliations:** 1 Department of Endocrinology of Portuguese Oncology Institute of Porto (IPO-Porto) & Clinical Research Unit—Research Center of IPO-Porto, 4200-072 Porto, Portugal; anapaulasantos@ipoporto.min-saude.pt (A.P.S.); isabel.torres@ipoporto.min-saude.pt (I.T.); 2 Department of Public Health and Forensic Sciences and Medical Education, Unit of Clinical Epidemiology, Predictive Medicine and Public Health, University of Porto Medical School, 4200-319 Porto, Portugal; acsantos@med.up.pt; 3 EPIUnit—Instituto de Saúde Pública, Universidade do Porto, 4050-600 Porto, Portugal; clara.castro@ipoporto.min-saude.pt (C.C.); luisraposoendo@gmail.com (L.R.); 4 Department of Epidemiology of Portuguese Oncology Institute of Porto (IPO-Porto), 4200-072 Porto, Portugal; 5 Endocrine, Cardiovascular & Metabolic Research, Unit for Multidisciplinary Research in Biomedicine (UMIB), University of Porto, 4050-313 Porto, Portugal; sspereira@icbas.up.pt (S.S.P.); helenacardoso@icbas.up.pt (H.C.); 6 Department of Anatomy, Institute of Biomedical Sciences Abel Salazar (ICBAS), University of Porto, 4050-313 Porto, Portugal; 7 Department of Pathology of Portuguese Oncology Institute of Porto (IPO-Porto) & Cancer Biology and Epigenetics Group—Research Center of IPO-Porto, 4200-072 Porto, Portugal; rmhenrique@icbas.up.pt; 8 Department of Pathology and Molecular Immunology of Institute of Biomedical Sciences Abel Salazar (ICBAS), University of Porto, 4050-313 Porto, Portugal; 9 Department of Endocrinology, Centro Hospitalar Universitário do Porto, 4099-001 Porto, Portugal

**Keywords:** gastroenteropancreatic neuroendocrine tumor, abdominal obesity, metabolic syndrome, glucose abnormalities

## Abstract

The determinants for gastroenteropancreatic neuroendocrine tumors (GEP-NET) recent burden are matters of debate. Obesity and metabolic syndrome (MetS) are well established risks for several cancers even though no link with GEP-NETs was yet established. Our aim in this study was to investigate whether well-differentiated GEP-NETs were associated with obesity and MetS. Patients with well-differentiated GEP-NETs (*n* = 96) were cross-matched for age, gender, and district of residence with a control group (*n* = 96) derived from the general population in a case-control study. Patients presented gastro-intestinal (75.0%) or pancreatic (22.9%) tumors, grade G1 (66.7%) or G2 (27.1%) with localized disease (31.3%), regional metastasis (16.7%) or distant metastasis (43.8%) at diagnosis, and 45.8% had clinical hormonal syndromes. MetS was defined according to Joint Interim Statement (JIS) criteria. Well-differentiated GEP-NETs were associated with MetS criteria as well as the individual components’ waist circumference, fasting triglycerides, and fasting plasma glucose (*p* = 0.003, *p* = 0.002, *p* = 0.011 and *p* < 0.001, respectively). The likelihood of the association was higher when the number of individual MetS components was greater than four. MetS and some individual MetS components including visceral obesity, dyslipidemia, and increased fasting glucose are associated with well-differentiated GEP-NET. This data provides a novel insight in unraveling the mechanisms leading to GEP-NET disease.

## 1. Introduction

Gastroenteropancreatic neuroendocrine tumors (GEP-NETs) are considered a rare entity even though a 6.5-fold increase in incidence was observed in the past four decades [1], which are believed to be predominantly driven by the rising number of the incidental detection of low-stage tumors [2]. GEP-NETs are currently the second most frequent digestive tumor only surpassed by colorectal cancer [3]. Grounded on the increasing knowledge related to the biology of the tumors accumulated in the past two decades, a great effort has been made in order to establish guidelines for GEP-NETs classification and management [4]. Nevertheless, despite the fact that significant advances were made towards the understanding of the genetics and molecular mechanisms associated with NETs, very little is known about the etiology of sporadic tumors or the reasons for the rising incidence observed over the past several decades [5].

The possible link between obesity and cancer was first described in the 1940s even though the molecular mechanisms underlying this association were only recently described [6,7]. Obesity is frequently associated with insulin resistance (IR), which is related to a state of systemic and local low grade chronic inflammatory state responsible for the activation of a number of signaling pathways involving hormone control, cell proliferation, and immunity [6,7] that led to neoplastic transformation of cells.

Insulin resistance (IR), metabolic syndrome (MetS), and type 2 diabetes mellitus (T2DM) are now well-established risk factors for many cancers including postmenopausal breast cancer, endometrial cancer, colorectal cancer, and hepatocarcinoma [8]. Chronic inflammation is also a well-recognized cancer promoter [9] such as chronic pancreatitis that leads to pancreatic cancer [10], ulcerative colitis to colon cancer [11], and non-alcoholic steatohepatitis (NASH) for liver cancer [12]. 

Whether obesity and MetS could be involved in the etiology of GEP-NETs to the extent of justifying the recent burden of the disease is unknown. This applies in particular to well-differentiated (WD) GEP-NETs, corresponding to the World Health Organization (WHO) 2010 grade G1 and G2, which have a natural history dramatically different from G3 poor-differentiated neuroendocrine carcinoma (NEC) [13].

Thus, the aim of the current study was to evaluate the possible association between MetS and MetS individual components with WD GEP-NETs by performing a case-control study comparing data from patients from a large tertiary cancer center with a matched control group derived from the background general population.

## 2. Results

### 2.1. Patients’ Characteristics

Table 1 provides the demographic, anthropometric, and clinical features of WD GEP-NET patients and controls. Patients’ mean age at WD GEP-NETs diagnosis was 58.2 years and 62.4 years at the time of a study assessment. There was a slight preponderance of males (52.1%) and the majority of the patients lived within the area of our institution (45.8%). Most patients had previous diagnosis of hypertension (63.5%), dyslipidemia (62.3%), or T2DM (17.7%). Family history of T2DM was present in 48.1% of cases. A large percentage of patients were under blood pressure lowering drugs (50.5%), lipid lowering medications (37.9%), statins (91.7%), and glucose lowering therapy (14.2%) including dipeptidyl peptidase-4 (DPP-4) inhibitors and/or metformin (58.3%), sulfonylureas (16.7%), or insulin (25.0%). Although there was no significant difference between WD GEP-NET patients and controls concerning the use of glucose lowering therapy, the proportion of patients under BP or lipid lowering therapy was significantly higher in patients than in controls (*p* < 0.001). There were no significant differences between patients and controls concerning weight, body mass index (BMI), systolic blood pressure (SBP), diastolic blood pressure (DBP), fasting plasma insulin (FPI), and Homeostasis Model Assessment Insulin Resistance (HOMA-IR). Total cholesterol (TC) and LDL-cholesterol (LDL-c) levels were significantly higher (*p* = 0.02 and *p* < 0.001, respectively) and HDL-c was significantly lower (*p* = 0.001) in controls when compared to patients. Fasting plasma glucose (FPG) was significantly higher in patients than in controls (*p* < 0.001) despite the fact that 14.2% of the patients were under glucose lowering therapy.

Subgroup analysis of patients comparing those that were under somatostatin analogues (SA) treatment with those that were not (Table 2) did not show any significant differences between the two groups regarding MetS (*p* = 0.746), WC (*p* = 0.198), TG levels (*p* = 0.503), HDL-c (*p* = 0.786), FPG (*p* = 0.862), FPI (*p* = 0.187), and HOMA-RI (*p* = 0.438).

The most frequent localization of the primary tumor was gastrointestinal (GI-NETs) in 75% of cases (60.0% in the ileum, 40% non-ileum), which is followed by pancreatic NETs (pNETs) that represented 22.9% of cases while, in two cases, the PT localization was unknown. The tumor’s hormone secretion profile was determined in the majority of the patients (90.6%) while 45.8% were found to be secreting tumors presenting with carcinoid syndrome (93.2%) or sporadic gastrinomas (6.8%). WD GEP-NETs were either grade G1 (66.7%) or G2 (27.1%) tumors. At presentation, 43.8% of patients were found to have distant metastasis, 16.7% of patients had loco-regional disease, and 31.2% of patients had localized disease, which included duodenal and colorectal NET polyps. Patients without distant metastasis referred to our center after surgical removal of the PT without information concerning available lymph nodes were considered to have an undetermined tumor stage (*n* = 8). WD GEP-NETs patients were treated in accordance with established treatment guidelines with SA (62.5%), liver ablative therapies including hepatic arterial embolization (TAE), radiofrequency (RF) and thermal ablation (TA) (29.5%), or with Peptide Receptor Radio Nuclide Therapy (PRRNT) with 177Luthetium-DOTATATE in 7.0%. Only one of the patients included was submitted to chemotherapy and no patients went on target therapies (Table 3).

### 2.2. WD GEP-NETs Association with Obesity, Glucose Abnormalities, MetS, and IR

A strong association between WD GEP-NETs and MetS (*p* = 0.003) and MetS individual Joint Interim Statement (JIS) criteria such as WC (*p* = 0.002), fasting TG (*p* = 0.011), FPG (*p* < 0.001), and a moderate association with severe IR (*p* = 0.014) was found (Table 4).

**Figure 1:**
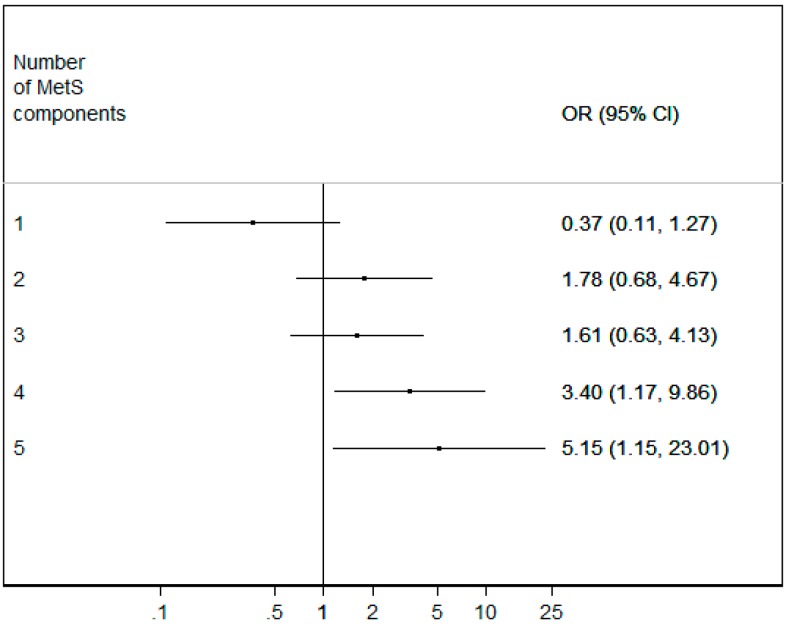
Tumor risk depending on the presence of different numbers of individual MetS components. WD GEP-NETs (well-differentiated gastro-enteric-pancreatic neuroendocrine tumors), OR (odds ratio), CI (confidence interval), and MetS (Metabolic Syndrome).

Moreover, the association increased significantly if four or five MetS individual components were present (*p* = 0.024 and *p* = 0.032, respectively) (Figure 1).

No association was found between WD GEP-NETs and BMI categories (*p* = 0.851 for excess weight and *p* = 0.847 for obesity) or the presence of T2DM (*p* = 0.608) even though IFG was significantly more frequent in patients than in controls (*p* = 0.013).

## 3. Discussion

Obesity and MetS are well established risk factors for several cancers even though whether there is a link between these conditions and the recent burden of GEP-NETs is yet to be confirmed. The aim of this study was to investigate whether there was an association between WD GEP-NETs and the anthropometric and metabolic abnormalities that characterize MetS.

Our results show that WD GEP-NETs are associated with MetS and some of the MetS individual components including elevated WC as surrogate for visceral obesity, fasting TG, and FPG. Moreover, the association was significantly increased if four or five individual MetS components were present. These findings also suggest WD GEP-NETs could also be associated with visceral obesity and severe IR despite the fact that no clear association with obesity grade or T2DM was found. Therefore, this data proposes that poor metabolic health, characterized by visceral obesity with altered glucose and lipid metabolism, are the most likely risk determinants of WD GEP-NETs. Similar association profiles were also described for other types of cancers including colon and rectal cancer [14], prostate cancer [15], esophageal cancer [16], and even thyroid cancer [17].

One of the main strengths of this study was enrolling a reasonably large patient sample with consistent data retrieval. All clinical and anthropometrical parameters were collected by the same researcher for what is considered a rare disease. Matching controls for age, gender, and the area of residence derived from the same background population ensured that these variables were similarly distributed in both groups. 

However, some limitations must be acknowledged. First, this was a single center-based case-control study. Additionally, due to the tertiary nature of our referral center, the PT removal and SA treatment initiation had already occurred when first observed at our institution in a considerable proportion of patients. In these circumstances, data was obtained retrospectively to reassure patient status before treatment. The sole exception was for FPI and FPG assessment that were performed while on SA to minimize the hyperglycemic effect of the treatment sampling that was made immediately before the next dosing [18].

Furthermore, as ongoing therapies were not subjected to match-control, the proportion of patients under BP or lipid lowering therapy was significantly higher in WD-GEP-NETS patients than in controls. This fact is unsurprising since subjects included in the control group were attended by general practitioners while patients with NETs were attended at a tertiary center where treatment intensification is more likely to occur. However, this dissimilarity between the groups should be interpreted into context because, according to the established JIS criteria for MetS of the International Diabetes Federation Task Force on Epidemiology and Prevention, ongoing treatment for any of the individual parameters is considered equivalent to the positive individual criteria regardless of the glucose, lipid, or blood pressure observed. Second, although the majority of patients under lipid lowering therapies, were already under treatment when first observed, these therapies were mainly statins (91.7%), which target mostly TG and LDL-C, which is less likely to interfere with triglycerides and HDL-C levels and bias MetS syndrome individual criteria. Third, the fact that a larger percentage of patients with NETs were under anti-hypertensive for a similar blood pressure profile further suggests the dissimilarity between the MetS risk profile between the two groups. 

Additional potential confounding factors such as a family history of cancer, cigarette smoking, alcohol consumption, dietary habits, physical activity, occupation, and socioeconomic status were not evaluated.

GEP-NETS were traditionally considered rare tumors. This paradigm has been changing over the last four decades since a nearly seven-fold increase in GEP-NETS incidence was registered with a current prevalence of 6.4 cases/100,000 inhabitants, which renders the ranking of the second most prevalent digestive neoplasia after colorectal cancer [1,3,5,19]. The reasons for the upsurge in GEP-NETS have been mostly attributed to an increase in incidental discovery by the widespread use of imaging techniques and improved medical skills while the actual mechanisms leading to the recent burden have not attracted extensive investigation and remains largely unknown. Nonetheless, epidemiological trends analysis using national statistics from several countries suggest that, to be able to explain the difference in geographic and ethnic incidence patterns, both genetic and environmental factors must be involved in the natural history of NETs [20].

Obesity has been known to be associated with cancer since the fourth decade of the 20th century [21]. More recently, mechanisms that link obesity and cancer were also established and particularly visceral adiposity was found to be linked with an increased risk of cancer independently of BMI [22]. Given to the rarity and heterogeneity of GEP-NETs, epidemiological studies designed to investigate the association between metabolic risk factors for the disease are lacking. Although obesity is not yet an established risk factor for GEP-NETS, few studies demonstrated that BMI increases pancreatic NET risk. A meta-analysis published in 2016 [23] describes two case-control studies linking BMI and pNETs [24,25] with a pool risk of 1.37 (95% CI 0.25 to 7.69, *p* < 0.001). The prevalence of incidental gastric NETs in obesity surgery candidates was found to be high [26] and the occurrence of a pNET co-secreting GLP-1 and glucagon in a patient previously submitted for gastric bypass surgery was also reported [27]. Although our data does not support an association between overweight or obesity with WD GEP-NETs, visceral obesity as assessed by the WC criteria for MetS was associated with an increased risk for WD GEP-NETs.

Few studies have addressed the putative association between glucose abnormalities with NETs and the majority refers to pNETs. Diabetes is a hallmark of some rare functioning (RF) GEP-NET such as glucagonomas, vasoactive intestinal polypeptide secreting tumors (VIPomas), and somatostatinomas and is present in 70% of non-functioning pNETS [28]. Moreover, hyperglycemia can also be a side effect of chemotherapy, SA, everolimus, and more recently PRRNT [18]. Our results show that not only patients with pancreatic NETs but also GI-NETs especially small bowel have a higher prevalence of MetS and glucose metabolism abnormalities. The present study points to a strong association between all sites WD GEP-NETs and IFG even before the initiation of treatments that can cause altered glucose homeostasis. This association was not exclusive of pNETs since it was also found in GI-NETs. No RF GEP-NET characterized by hyperglycemia were included in this cohort. A strong association between diabetes and pNETs with an estimate effect of 2.76 (95% CI 1.65–4.64, *p* = 0.090) was formerly found in three case-control studies [24,25,29]. This effect was even higher in cases with recent onset diabetes (OR 12.80, 95%CI 2.47–66.42, *p* = 0.135) and insulin treated patients (OR 4.80, 95% CI 1.20–18.90). Two studies previously described the association between diabetes and tumors other than pNETs. In women with pre-existing T2DM, gastric endocrine tumors (especially T1-GET) and small bowel NETs were found to be increased seven-fold and two-fold, respectively [24]. Increased prevalence of impaired glucose tolerance in patients with serotonin secreting metastatic NETs when compared to non-secreting tumors was initially reported in 1975 [30]. Moreover, a recent publication from Valente et al. concluded that non-recent diabetes was associated with an increased occurrence of pNETs especially in metastatic disease and an advanced grade [31]. 

Our findings also support that there is an association of MetS with WD GEP-NETs. There is accumulating evidence that visceral obesity, insulin resistance, hyperinsulinemia, chronic inflammation, and T2DM can lead to increased cell proliferation, apoptosis inhibition, angiogenesis, and impaired immunity [32,33]. MetS is a cluster of risk factors with a well-established association with cardiovascular disease that was also demonstrated to be a modifiable risk factor for several cancers [34] such as breast cancer in postmenopausal women (HR 1.89, 95% CI 1.29–2.77) [35]. Two studies from South Korea concluded that there is an association between MetS and rectal NETs (r-NETs) (OR 1.768, 95% CI 1.071–2.918, *p* = 0.026) [36,37].

In the present study, no significant differences in FPI and HOMA-IR were found between patients and controls. Nonetheless, the proportion of severe IR (HOMA-IR ≥ 5) was significantly higher in patients than in controls. Despite a large proportion of patients being under SA at the time of FPI and an FG determination (60%), no differences in MetS criteria, MetS individual components, FPI, HOMA-RI, and the proportion of insulin resistant and severe insulin resistant patients were found between patients under SA treatment or were untreated, which suggests that our findings were not influenced by SA (Table 2).

Our results also show that, although no differences were found in median TG levels between patients and controls, the proportion of GEP-NET patients with TG ≥ 150 mg/dL was significantly higher than in controls (*p* = 0.011). Despite the fact that low HDL-c was identified as an independent risk factor for r-NETs in a South Korean cohort (OR 1.85, 95% CI 1.10–3.11, *p* = 0.021) [36,37], the unexpected finding of lower TC and c-LDL levels as well as higher c-HDL levels in our patients’ cohort compared to controls could be attributed to treatment intensification of patients with GEP-NETs when compared to the general population since 37.2% of the patients vs. 9.9% of controls were under drug treatment for dyslipidemia. Previously, only hypercholesterolemia was found to be a risk factor for rectal GEP-NETs (OR 1.007, 95% CI 1.001–1.013; *p* = 0.016) in a single study [36]. This is in contrast with hypertension since no association was found between hypertension and WD GEP-NETs.

## 4. Materials and Methods

Patients with confirmed WD GEP-NETs (*n* = 96) were recruited from the endocrine tumors clinic of a large tertiary referral center for oncological diseases. The inclusion criteria were a confirmed diagnosis of WD GEP-NETs by histopathology and/or PET-68Ga-DOTA-NOC. The exclusion criteria were under 18 years of age when first diagnosed, familial GEP-NETs, NEC, and type 1 gastric endocrine tumor (T1-GET) since these tumors have well-established etiology and distinctive behavior [13,38,39].

From a total number of patients recruited with confirmed WD GEP-NETs (*n* = 120) that consented to participate in the study, those who did not fulfil the inclusion criteria or had insufficient data for analysis were excluded (*n* = 24). The remainder of patients (*n* = 96) were then matched for age, gender, and district of residence with a control group (*n* = 96) of the general population derived from the PORMETs study, which is a nationwide epidemiological study designed to assess the prevalence of MetS in the general population [40,41,42]. The present study was approved by the National Data Protection Committee (CNPD 4906/2015) as well as the Institutional Ethics Committee (IPO 366/2013). Patients gave their written informed consent to participate and were consecutively enrolled as attending routine clinic appointments.

Data for analysis was collected through a face-to-face patient interview to assess the past medical history of T2DM, hypertension, dyslipidemia, ongoing medications, and family history of T2DM while height, weight, waist circumference (WC), and blood pressure (BP) measurements were collected directly or indirectly, according to medical practice standards. Most patients were newly diagnosed WD GEP-NETs patients who were referred to our center and the parameters used for the assessment of metabolic syndrome refer to the time of diagnosis. For patients with longer disease duration referred to our center after treatment initiation (surgery or somatostatin analogues), data was retrieved from patient digital records from other institutions (hospital or general practice registries) to ensure a minimum bias.

Biochemical data including FPG and the lipid profile were evaluated while off any active anti-tumor treatment. The only exception was for FPG and FPI measurements that were used for HOMA-IR calculation, which were assessed while on somatostatin analogues (SA) in those patients who were already under oncological treatment. WD GEP-NETs were classified according to primary tumor localization, the presence of the hormone secretion syndrome, the WHO 2010 grading system, and disease extension (ENETS TNM staging system) [43,44]. Cases with insufficient data to allow grading were classified as WD GEP-NET if found to express somatostatin receptors on PET-68Ga-DOTA-NOC (*n* = 6). Patients with metastatic tumors and carcinoid syndrome without any visible pancreatic or thoracic lesions on imaging investigations were classified as having WD GEP-NET with an occult primary tumor (*n* = 2). No insulinoma or rare functional pancreatic NET presenting with hyperglycemia such as glucagonoma, VIPoma or somatostatinoma were included in this study series.

Patients were classified into three categories according to the BMI, which included normal weight (BMI < 25 Kg/m^2^), overweight (BMI 25–29.9 Kg/m^2^), or obese (BMI ≥ 30 Kg/m^2^) [45] and according to FPG levels into normoglycemic (NG, FPG < 100 mg/dL) and impaired fasting glucose (IFG, FPG 100–126 mg/dL) or T2DM (T2DM, FPG ≥ 126 mg/dL) [46]. MetS was classified, according to the Joint Interim Statement (JIS) of NHLBI/AHA/WHF/IAS/IASO criteria [47]: WC ≥ 88 cm (female) or 102 cm (male), BP ≥ 130 mmHg and/or 85 mmHg or previous history of high BP or under BP lowering medication. HDL-cholesterol (HDL-c) < 40 mg/dL (male) or ≤50 mg/dL (female) drug treatment for reduced HDL-c, triglycerides (TG) ≥ 150 mg/dL or under triglyceride lowering drugs, and FPG ≥ 100 mg/dL or ongoing glucose-lowering drug treatments.

Insulin was determined by an automated enzyme-labeled chemiluminescent immune metric solid-phase assay (IMMULITE 2000). IR was assessed by HOMA-IR index calculated using the formula FPI (μU/mL)/FPG (mg/dL)/405 [19]. IR cut-offs were based on Matthews [48] definition: <3 (insulin sensitive), ≥ 3 < 5 (IR) and ≥ 5 (severe IR).

Statistical analysis was performed using PASW 18.0. Categorical and continuous variables were summarized using descriptive statistics (frequencies for categorical, mean/standard deviation or median/interquartile range for continuous, as appropriate). Proportions were compared by the Chi-squared or Fisher Exact test. Means were compared using the t-test or ANOVA while medians were compared using the Mann-Whitney or Kruskal-Wallis tests. Unconditional logistic regression models were used to evaluate the odds of developing GEP-NET, according to weight, glucose abnormalities, IR, and MetS criteria. A level of significance of 0.05 was adopted.

## 5. Conclusions

In conclusion, our findings show that WD GEP-NETS are associated with MetS, elevated WC, elevated FPG, elevated TG, and severe IR. These results are a breakthrough toward understanding the recent WD GEP-NET “epidemic” since the association of the anthropometric, clinical, and biochemical abnormalities that characterize MetS or IR with these specific tumors, according to the primary location, the hormonal functional status, and grading or staging that had not been previously reported. Although requiring confirmation in larger scale studies, these novel findings could provide crucial insight toward the understanding of putative mechanisms leading to disease and prove important to establish targeted preventive and treatment interventions [49] by addressing cancer as a metabolic disease [50].

## Figures and Tables

**Table 1:** Demographic, anthropometric, clinical, and biochemical features of patients with WD GEP-NETs and controls.

Demographic and Clinical Features	Patients (*n* = 96)	Controls (*n* = 96)	*p*
Age in years—mean (SD)	62.4 (11.20)	62.4 (12.1)	0.979
Age at Diagnosis in years—mean (SD)	58.2 (11.2)	-	-
Duration of the disease in months—mean (SD), (*n* = 92)	55.3 (37.5)	-	-
Gender—*n* (%)			
Male	50 (52.1)	52 (54.2)	0.772
Female	46 (47.9)	44 (45.8)	
**Metabolic Treatment**			
Previous anti-hypertensive treatment (*n* = 95/71)	48 (50.5)	12 (16.9)	<0.001
Previous anti-dyslipidemia treatment (*n* = 95/71)	36 (37.9)	7 (9.9)	<0.001
Statins	33 (91.7)	6 (8.5)	-
Fibrates	3 (8.3)	3 (3.2)	-
Previous anti-diabetic treatment (*n* = 79)	12 (14.2)	3 (4.2)	0.102
Insulin sensitizers	7 (58.3)	3 (4,2)	
Sulfonylureas	2 (16.7)	-	-
Insulin	3 (25.0)	-	-
**Clinical Evaluation**			
Height, cm—median (IQR)	164.0 (14.5)	163.0 (39,0)	0.573
Weight, cm—mean (SD)	72.6 (13.6)	72.0 (13.3)	0.753
BMI, Kg/m^2^—mean (SD)	26.9 (4.2)	27.2 (4.1)	0.645
WC, cm—mean (SD)	94.9 (12.0)	93.0 (10.6)	0.236
SBP, mmHg—median (IQR)	135.0 (21.0)	130.0 (28.0)	0.247
DBP, mmHg—median (IQR)	75.5 (17.0)	70.5 (12.0)	0.203
**Biochemical Evaluation**			
TC, mg/dL—mean (SD)	192.1 (44.4)	208.1 (49.8)	0.020
LDL-c, mg/dL—mean (SD)	114.1 (37.1)	139.6 (41.0)	<0.001
HDL-c, mg/dL—mean (SD)	50.8 (13.1)	44.8 (12.3)	0.001
TG, mg/dL—median (IQR)	117.5 (78.5)	105.0 (77)	0.091
FPG, mg/dL—median (IQR)	101.0 (22.0)	88.5 (27.5)	<0.001
FPI—median (IQR)	6.2 (5.0)	5.8 (6.0)	0.372
HOMA-R—median (IQR)	1.4 (1.6)	1.4 (1.6)	0.274

BMI (Body Mass Index), WC (waist circumference), SBP (systolic blood pressure), DBP (diastolic blood pressure), TC (total cholesterol), TG (Triglycerides), FPG (fasting plasma glucose), FPI (fasting plasma insulin), HOMA-IR (homeostasis model assessment insulin resistance), BMI (body mass index).

**Table 2:** Comparison of anthropometric and biochemical metabolic profile of WD GEP-NET patients under somatostatin analogues (SA positive) treatment versus patients with no somatostatin analogue exposure (SA negative).

Clinical Features	SA Positive (*n* = 60)	SA Negative (*n* = 36)	*p*
WC (mean/SD)	96.2 (12.4)	96.7 (11.3)	0.198
TG (median (IQR))	121.5 (73.3)	111.0 (91.5)	0.503
HDL (median (IQR))	50.6 (13.3)	55.7 (12.9)	0.786
FPG (median (IQR))	102.0 (22.0)	99.5 (20.0)	0.862
FPI (median (IQR))	6.1 (4.0)	7.3 (9.0)	0.187
HOMA-R (median (IQR))	1.4 (0.8)	1.5 (2.1)	0.438

WC (waist circumference), TG (Triglycerides), Fasting Glucose (FPG), FPI (fasting plasma insulin), HOMA-IR (Homeostasis Model Assessment Insulin Resistance).

**Table 3:** WD GEP-NETs patient characteristics.

Localization of PT (*n* = 96)	*n* (%)
GI-NET	72 (75.0)
Jejunum-ileum	45 (62.5)
Duodenum	10 (13.9)
Rectum	8 (11.1)
Appendix	5 (6.9)
Colon	2 (2.8)
Stomach	1 (1.4)
Ampulla	1 (1.4)
p-NET	22 (22.9)
Unknown (UK)	2 (2.1)
**Hormonal Syndrome (*n* = 96)**	
Yes (93.2% carcinoid syndrome; 6.8% gastrinomas)	44 (45.8)
No	43 (44.8)
Unknown (UK)	9 (9.4)
***Grading* (WHO 2010)—*n* = 96**	
NETG1	64 (66.7)
NETG2	26 (27.1)
Unknown (UK)	6 (6.3)
***Staging* (ENETS)—(*n* = 96)**	
Local disease	30 (31.3)
Loco regional disease	16 (16.7)
Disseminated disease	42 (43.8)
Unknown (UK)	8 (8.3)
**Past History**	
Family History of T2-DM (*n* = 81)	39 (48.1)
Hypertension (*n* = 96)	61 (63.5)
Dyslipidemia (*n* = 96)	60 (62.5)
T2DM (*n* = 96)	17 (17.7)
**NET Treatment**	
Endoscopic therapy (*n* = 95)	11 (11.6)
Surgery (*n* = 96)	73 (76.8)
SA (*n* = 95)	60 (62.5)
Liver ablative therapies (*n* = 95)	28 (29.5)
PRRNT (*n* = 95)	7 (7.4)
Chemotherapy (*n* = 96)	1 (1.0)
Target therapies (*n* = 96)	0 (0.0)

**Table 4:** Association of MetS, MetS components, and IR with WD GEP-NETs and controls.

Clinical Features	Pts. *n* (%)	Controls *n* (%)	OR (95% CI)	*p*
**Obesity Classification**				
Normal weight (BMI < 25 Kg/m^2^)	31 (32.3)	33 (34.4)	1	
Excess weight (25 ≤ BMI < 30 Kg/m^2^)	41(42.7)	41 (42.7)	1.1 (0.6–2.0)	0.851
Obesity (BMI ≥ 30Kg/m^2^)	24 (25.0)	22 (22.9)	1.2 (0.5–2.5)	0.847
**Classification of Glucose Abnormalities**				
Normal	62 (64.6)	71 (75.5)	1	
IFG	14 (14.6)	4 (4.3)	4.0 (1.3–12.8)	0.013
T2DM	20 (20.8)	19 (20.2)	1.2 (0.6–2.5)	0.608
**Metabolic Syndrome and Components**				
WC ≥ 80 (F)/94 (M) cm	55 (58.9)	34 (35.8)	2.5 (1.4–4.6)	0.002
BP ≥ 130/85 mmHg (or anti-hypertensive drugs)	63 (65.6)	61 (64.2)	1.06 (0.6–1.9)	0.838
C-HDL < 50 (F)/40 (M) mg/dL (or anti-*dyslipidemia* drugs)	52 (54.2)	48 (50.5)	1.6 (0.7–2.0)	0.615
TG ≥ 150 mg/dL (or anti-dyslipidemia drugs)	41(42.7)	24 (25.3)	2.2 (1.2–4.1)	0.011
FPG ≥ 100 mg/dL (or hypoglycemic drugs)	53 (55.2)	21 (22.1)	4.3 (2.3–8.2)	< 0.001
Metabolic syndrome	58 (60.4)	37 (54.4)	2.4 (1.3–4.3)	0.003
**IR Classification *n* (%)**				
Insulin sensitive (HOMA-IR < 3)	54 (56.3)	80 (85.1)	1	
Insulin resistant (3 ≤ HOMA-IR < 5)	2 (3.0)	10 (10.6)	0.3 (0.1–1.4)	0.131
Very insulin resistant (HOMA-IR ≥ 5)	11 (11.5)	4 (4.3)	4.1 (1.2–13.5)	0.014

WC (waist circumference), BP (blood pressure), TG (triglycerides), FPG (Fasting Plasma Glucose)), Metabolic Syndrome (Metabolic Syndrome), BMI (Body Mass Index), IFG (Impaired Fasting Glucose), T2DM (type 2 diabetes mellitus), IR (insulin resistance), HOMA-IR (Homeostasis Model Assessment Insulin Resistance).

## References

[1] Dasari A., Shen C., Halperin D., Zhao B., Zhou S., Xu Y., Shih T., Yao J.C. (2017). Trends in the incidence, prevalence, and survival outcomes in patients with neuroendocrine tumors in the united states. JAMA Oncol..

[2] McMullen T., Al-Jahdali A., de Gara C., Ghosh S., McEwan A., Schiller D. (2017). A population-based study of outcomes in patients with gastrointestinal neuroendocrine tumours. Can. J. Surg..

[3] Yao J.C., Hassan M., Phan A., Dagohoy C., Leary C., Mares J.E., Abdalla E.K., Fleming J.B., Vauthey J.N., Rashid A. (2008). One hundred years after “carcinoid”: Epidemiology of and prognostic factors for neuroendocrine tumors in 35,825 cases in the United States. J. Clin. Oncol..

[4] O’Toole D., Kianmanesh R., Caplin M. (2016). Enets 2016 consensus guidelines for the management of patients with digestive neuroendocrine tumors: An update. Neuroendocrinology.

[5] Pavel M., de Herder W.W. (2017). Enets consensus guidelines for the standard of care in neuroendocrine tumors. Neuroendocrinology.

[6] Byers T., Sedjo R.L. (2015). Body fatness as a cause of cancer: Epidemiologic clues to biologic mechanisms. Endocr. Relat. Cancer.

[7] Vigneri P., Frasca F., Sciacca L., Pandini G., Vigneri R. (2009). Diabetes and cancer. Endocr. Relat. Cancer.

[8] Arcidiacono B., Iiritano S., Nocera A., Possidente K., Nevolo M.T., Ventura V., Foti D., Chiefari E., Brunetti A. (2012). Insulin resistance and cancer risk: An overview of the pathogenetic mechanisms. Exp. Diabetes Res..

[9] Rakoff-Nahoum S. (2006). Why cancer and inflammation?. Yale J. Biol. Med..

[10] Gukovsky I., Li N., Todoric J., Gukovskaya A., Karin M. (2013). Inflammation, autophagy, and obesity: Common features in the pathogenesis of pancreatitis and pancreatic cancer. Gastroenterology.

[11] Scarpa M., Castagliuolo I., Castoro C., Pozza A., Scarpa M., Kotsafti A., Angriman I. (2014). Inflammatory colonic carcinogenesis: A review on pathogenesis and immunosurveillance mechanisms in ulcerative colitis. World J. Gastroenterol..

[12] Dongiovanni P., Romeo S., Valenti L. (2014). Hepatocellular carcinoma in nonalcoholic fatty liver: Role of environmental and genetic factors. World J. Gastroenterol..

[13] Heetfeld M., Chougnet C.N., Olsen I.H., Rinke A., Borbath I., Crespo G., Barriuso J., Pavel M., O’Toole D., Walter T. (2015). Characteristics and treatment of patients with g3 gastroenteropancreatic neuroendocrine neoplasms. Endocr. Relat. Cancer.

[14] Kim N.H., Jung Y.S., Park J.H., Park D.I., Sohn C.I. (2018). Influence of obesity and metabolic abnormalities on the risk of developing colorectal neoplasia. Dig. Dis. Sci..

[15] Chen Z., Deng J., Yan Y., Li M., Chen C., Chen C., Zhao S., Song T., Liu T., Wen X. (2018). Risk analysis of prostate cancer treatments in promoting metabolic syndrome development and the influence of increased metabolic syndrome on prostate cancer therapeutic outcome. Horm. Cancer.

[16] Liu B., Cheng B., Wang C., Chen P., Cheng Y. (2018). The prognostic significance of metabolic syndrome and weight loss in esophageal squamous cell carcinoma. Sci. Rep..

[17] Yin D.T., He H., Yu K., Xie J., Lei M., Ma R., Li H., Wang Y., Liu Z. (2018). The association between thyroid cancer and insulin resistance, metabolic syndrome and its components: A systematic review and meta-analysis. Int. J. Surg..

[18] Verges B., Walter T., Cariou B. (2014). Endocrine side effects of anti-cancer drugs: Effects of anti-cancer targeted therapies on lipid and glucose metabolism. Eur. J. Endocrinol..

[19] Oberg K., Knigge U., Kwekkeboom D., Perren A., Group E.G.W. (2012). Neuroendocrine gastro-entero-pancreatic tumors: Esmo clinical practice guidelines for diagnosis, treatment and follow-up. Ann. Oncol..

[20] Huguet I., Grossman A.B., O’Toole D. (2017). Changes in the epidemiology of neuroendocrine tumours. Neuroendocrinology.

[21] Tannenbaum A. (1940). Relationship of body weight to cancer incidence. Arch. Pathol..

[22] Dong Y., Zhou J., Zhu Y., Luo L., He T., Hu H., Liu H., Zhang Y., Luo D., Xu S. (2017). Abdominal obesity and colorectal cancer risk: Systematic review and meta-analysis of prospective studies. Biosci. Rep..

[23] Leoncini E., Carioli G., La Vecchia C., Boccia S., Rindi G. (2016). Risk factors for neuroendocrine neoplasms: A systematic review and meta-analysis. Ann. Oncol..

[24] Hassan M.M., Phan A., Li D., Dagohoy C.G., Leary C., Yao J.C. (2008). Risk factors associated with neuroendocrine tumors: A u.S.-based case-control study. Int. J. Cancer.

[25] Halfdanarson T.R., Bamlet W.R., McWilliams R.R., Hobday T.J., Burch P.A., Rabe K.G., Petersen G.M. (2014). Risk factors for pancreatic neuroendocrine tumors: A clinic-based case-control study. Pancreas.

[26] Al-Harbi O., Shakir M., Al-Brahim N. (2013). Gastric carcinoid and obesity: Association or coincidence? Report of two cases and literature review. Case Rep. Gastrointest. Med..

[27] Guimaraes M., Rodrigues P., Pereira S.S., Nora M., Goncalves G., Albrechtsen N.W., Hartmann B., Holst J.J., Monteiro M.P. (2015). Glp1 and glucagon co-secreting pancreatic neuroendocrine tumor presenting as hypoglycemia after gastric bypass. Endocrinol. Diabetes Metab. Case Rep..

[28] Vinik A.I., Gonzales M.R. (2011). New and emerging syndromes due to neuroendocrine tumors. Endocrinol. Metab. Clin. N. Am..

[29] Capurso G., Falconi M., Panzuto F., Rinzivillo M., Boninsegna L., Bettini R., Corleto V., Borgia P., Pederzoli P., Scarpa A. (2009). Risk factors for sporadic pancreatic endocrine tumors: A case-control study of prospectively evaluated patients. Am. J. Gastroenterol..

[30] Feldman J.M., Plonk J.W., Bivens C.H., Lebovitz H.E. (1975). Glucose intolerance in the carcinoid syndrome. Diabetes.

[31] Valente R., Hayes A.J., Haugvik S.P., Hedenstrom P., Siuka D., Korsaeth E., Kammerer D., Robinson S.M., Maisonneuve P., Delle Fave G. (2017). Risk and protective factors for the occurrence of sporadic pancreatic endocrine neoplasms. Endocr. Relat. Cancer.

[32] Jee S.H., Kim H.J., Lee J. (2005). Obesity, insulin resistance and cancer risk. Yonsei Med. J..

[33] Font-Burgada J., Sun B., Karin M. (2016). Obesity and cancer: The oil that feeds the flame. Cell Metab..

[34] Uzunlulu M., Telci Caklili O., Oguz A. (2016). Association between metabolic syndrome and cancer. Ann. Nutr. Metab..

[35] Agnoli C., Grioni S., Sieri S., Sacerdote C., Ricceri F., Tumino R., Frasca G., Pala V., Mattiello A., Chiodini P. (2015). Metabolic syndrome and breast cancer risk: A case-cohort study nested in a multicentre italian cohort. PLoS ONE.

[36] Pyo J.H., Hong S.N., Min B.H., Lee J.H., Chang D.K., Rhee P.L., Kim J.J., Choi S.K., Jung S.H., Son H.J. (2016). Evaluation of the risk factors associated with rectal neuroendocrine tumors: A big data analytic study from a health screening center. J. Gastroenterol..

[37] Jung Y.S., Yun K.E., Chang Y., Ryu S., Park J.H., Kim H.J., Cho Y.K., Sohn C.I., Jeon W.K., Kim B.I. (2014). Risk factors associated with rectal neuroendocrine tumors: A cross-sectional study. Cancer Epidemiol. Biomarkers Prev..

[38] Delle Fave G., O’Toole D., Sundin A., Taal B., Ferolla P., Ramage J.K., Ferone D., Ito T., Weber W., Zheng-Pei Z. (2016). Enets consensus guidelines update for gastroduodenal neuroendocrine neoplasms. Neuroendocrinology.

[39] Benafif S., Eeles R. (2016). Diagnosis and management of hereditary carcinoids. Recent Results Cancer Res..

[40] Raposo L., Severo M., Santos A.C. (2018). Adiposity cut-off points for cardiovascular disease and diabetes risk in the portuguese population: The pormets study. PLoS ONE.

[41] Raposo L., Martins S., Ferreira D., Guimaraes J.T., Santos A.C. (2017). Vitamin D, parathyroid hormone and metabolic syndrome - the pormets study. BMC Endocr. Disord..

[42] Raposo L., Severo M., Barros H., Santos A.C. (2017). The prevalence of the metabolic syndrome in portugal: The pormets study. BMC Public Health.

[43] Rindi G., Kloppel G., Alhman H., Caplin M., Couvelard A., de Herder W.W., Erikssson B., Falchetti A., Falconi M., Komminoth P. (2006). Tnm staging of foregut (neuro)endocrine tumors: A consensus proposal including a grading system. Virchows Arch..

[44] Rindi G., Kloppel G., Couvelard A., Komminoth P., Korner M., Lopes J.M., McNicol A.M., Nilsson O., Perren A., Scarpa A. (2007). Tnm staging of midgut and hindgut (neuro) endocrine tumors: A consensus proposal including a grading system. Virchows Arch..

[45] Borrell L.N., Samuel L. (2014). Body mass index categories and mortality risk in us adults: The effect of overweight and obesity on advancing death. Am. J. Public Health.

[46] American Diabetes Association (2010). Diagnosis and classification of diabetes mellitus. Diabetes Care.

[47] Alberti K.G., Eckel R.H., Grundy S.M., Zimmet P.Z., Cleeman J.I., Donato K.A., Fruchart J.C., James W.P., Loria C.M., Smith S.C. (2009). Harmonizing the metabolic syndrome: A joint interim statement of the international diabetes federation task force on epidemiology and prevention; national heart, lung, and blood institute; american heart association; world heart federation; international atherosclerosis society; and international association for the study of obesity. Circulation.

[48] Matthews D.R., Hosker J.P., Rudenski A.S., Naylor B.A., Treacher D.F., Turner R.C. (1985). Homeostasis model assessment: Insulin resistance and beta-cell function from fasting plasma glucose and insulin concentrations in man. Diabetologia.

[49] Anand P., Kunnumakkara A.B., Sundaram C., Harikumar K.B., Tharakan S.T., Lai O.S., Sung B., Aggarwal B.B. (2008). Cancer is a preventable disease that requires major lifestyle changes. Pharm. Res..

[50] Seyfried T.N., Shelton L.M. (2010). Cancer as a metabolic disease. Nutr. Metab. (Lond.).

